# Improved intensive care unit survival for critically ill allogeneic haematopoietic stem cell transplant recipients following reduced intensity conditioning

**DOI:** 10.1111/bjh.12294

**Published:** 2013-03-18

**Authors:** William M Townsend, Ailsa Holroyd, Rachel Pearce, Stephen Mackinnon, Prakesh Naik, Anthony H Goldstone, David C Linch, Karl S Peggs, Kirsty J Thomson, Mervyn Singer, David C J Howell, Emma C Morris

**Affiliations:** 1 Department of Haematology, University College London Hospitals NHS Foundation Trust and UCL Medical SchoolLondon, UK; 2 British Society for Blood and Marrow TransplantationLondon, UK; 3 Department of Haematology, Royal Free London NHS Foundation Trust and UCL Medical SchoolLondon, UK; 4 Department of Intensive Care Medicine, University College London Hospitals NHS Foundation Trust and UCL Medical SchoolLondon, UK; 5 Department of Immunology, Royal Free London NHS Foundation Trust and UCL Medical SchoolLondon, UK

**Keywords:** intensive care, allogeneic haematopoietic stem cell transplantation, transplant toxicity, long term outcome

## Abstract

The use of allogeneic haematopoietic stem cell transplantation (Allo-HSCT) is a standard treatment option for many patients with haematological malignancies. Historically, patients requiring intensive care unit (ICU) admission for transplant-related toxicities have fared extremely poorly, with high ICU mortality rates. Little is known about the impact of reduced intensity Allo-HSCT conditioning regimens in older patients on the ICU and subsequent long-term outcomes. A retrospective analysis of data collected from 164 consecutive Allo-HSCT recipients admitted to ICU for a total of 213 admissions, at a single centre over an 11·5-year study period was performed. Follow-up was recorded until 31 March 2011. Autologous HSCT recipients were excluded. In this study we report favourable ICU survival following Allo-HSCT and, for the first time, demonstrate significantly better survival for patients who underwent Allo-HSCT with reduced intensity conditioning compared to those treated with myeloablative conditioning regimens. In addition, we identified the need for ventilation (invasive or non-invasive) as an independently significant adverse factor affecting short-term ICU outcome. For patients surviving ICU admission, subsequent long-term overall survival was excellent; 61% and 51% at 1 and 5 years, respectively. Reduced intensity Allo-HSCT patients admitted to ICU with critical illness have improved survival compared to myeloablative Allo-HSCT recipients.

Allogeneic haematopoietic stem cell transplantation (Allo-HSCT) offers a chance of cure for many haematological malignancies. Standard myeloablative (MA) conditioning regimens are designed for maximal anti-tumour effect. Such approaches are associated with considerable morbidity and mortality due to treatment-related toxicities (overwhelming infection, end-organ failure and graft-versus-host disease [GvHD]), which increase with recipient age, extent of prior treatment, disease-related end-organ damage and concomitant diseases.

Transplant-related complications necessitate ICU admission in a significant proportion of patients undergoing Allo-HSCT (Naeem *et al*, 2006; Scales *et al*, 2008). Previously published survival rates for patients admitted to ICU following HSCT (both allogeneic and autologous) have been very poor with an in-hospital mortality rate of 54–96%, while the reported mortality rates for ventilated HSCT patients in these studies have been extremely high at 81–96% (Afessa *et al*, 1992; Faber-Langendoen *et al*, 1993; Rubenfeld & Crawford, 1996; Jackson *et al*, 1998; Paz *et al*, 1998; Price *et al*, 1998; Bach *et al*, 2001; Scales *et al*, 2008).

Following the introduction of reduced intensity (RI) conditioning regimens 20 years ago, there has been an expansion of allogeneic transplantation in older patients. RI regimens do not rely on cytotoxic measures to ablate recipient haematopoietic cells but are highly immunosuppressive. This permits the engraftment of donor-derived cells alongside residual recipient cells in a transient state of immunological tolerance. Such regimens commonly include fludarabine combined with monoclonal antibodies directed against T cells and/or low-dose total body irradiation (TBI). Removal of T cells from the donor stem cell preparation [T-cell depletion, (TCD)], to reduce GvHD with subsequent add-back of donor lymphocytes to provide anti-tumour immune responses, is also employed. Success has been demonstrated in a range of haematological malignancies (Morris & Mackinnon, 2005; Craddock, 2008; Thomson *et al*, 2010; Peggs *et al*, 2011). Such approaches have reduced the immediate transplant-related mortality (TRM) due to less toxic conditioning chemo/radiotherapy but, as a result of increased and prolonged immune suppression, infective complications may be more common in the post-transplant period (Martino *et al*, 2009). Such prolonged immune suppression in older adults, with worse pre-transplant co-morbidity scores, may be predicted to result in increased respiratory infections and/or ICU referral.

Many studies have evaluated the outcome of critically ill haematology patients, but only a few large studies have focussed solely on the outcome of Allo-HSCT recipients requiring ICU admission (Pène *et al*, 2006; Depuydt *et al*, 2011). In these studies, <10% of patients received RI conditioning and there are very limited published data directly comparing ICU outcomes for RI and MA Allo-HSCT recipients. This is increasingly important as, in the UK, the majority of allogeneic transplants now utilize RI conditioning (K Kirkland, British Society of Blood and Marrow Transplantation [BSBMT] Data Registry, Guy's Hospital, London, personal communication). In addition, there are only a few published studies considering the long-term survival of Allo-HSCT admitted to ICU, which reported survival at 1 year or beyond (Pène *et al*, 2006; Lim *et al*, 2007; Scales *et al*, 2008; Bokhari *et al*, 2010).

The primary aim of this study was to determine the short and long-term outcomes of patients admitted to the ICU in our institution following Allo-HSCT over an 11·5-year period. We assessed whether RI regimens were associated with improved ICU and long-term survival, and determined whether there were are any factors predictive of outcome.

## Materials and methods

### Patients and setting

A retrospective analysis of data collected from 164 consecutive adult and adolescent Allo-HSCT recipients admitted to the critical care unit for a total of 213 admissions, at a single centre, University College London Hospitals NHS Foundation Trust (UCLH), between June 1996 and December 2007 was performed. Follow-up was recorded until 31 March 2011. Autologous HSCT recipients were excluded. The critical care unit is mixed-dependency, caring for both level 2 (High Dependency Unit – HDU) and level 3 (ICU) patients. All Allo-HSCT patients admitted to this unit were included in the analysis. Intensive care physicians share responsibility of care with admitting transplant haematologists. The critical care unit routinely admits critically ill immuno-compromised patients and is the only location in the hospital offering organ support, such as mechanical ventilation, vasoactive drugs and renal replacement therapy.

### Data collection

The following demographic variables, haematological disease-related and transplant-related characteristics were recorded: age, sex and year of ICU admission, underlying haematological disease, conditioning regimen, GvHD prophylaxis, conditioning regimen intensity – myeloablative (MA) or reduced intensity (RI), and type of donor (sibling or unrelated). Factors studied in relation to the severity of illness requiring admission to ICU were: number of ICU admissions, reason for admission, number and modality of organ support, Acute Physiology and Chronic Health Evaluation (APACHE II) score, and length of ICU stay. Laboratory-based parameters on day of ICU admission analysed were: neutrophil count, platelet count, serum bilirubin and urea. Time from Allo-HSCT to ICU admission was recorded. Data were retrieved from patient case notes, the UK Intensive Care National Audit and Research Centre database and the UCLH Clinical Data Repository. Long-term follow-up data were obtained from transplant clinics, medical records, and primary care physicians.

### Definitions

The following terms were used as indications for admission to ICU and are defined below: sepsis, respiratory, renal, haemodynamic, neurological, acute respiratory distress syndrome, and acute lung injury. *Sepsis*: Sepsis was defined by the presence of a systemic inflammatory response syndrome (SIRS) with a concurrent documented or assumed infection (Bone *et al*, 2009). *Respiratory*: Respiratory failure included type I and type II failure. *Renal*: Renal admissions to ICU were defined by the need for renal replacement therapy. *Haemodynamic*: Admissions for the purposes of invasive cardiovascular monitoring with or without inotropic support were defined as haemodynamic. *Neurological*: Admissions with a Glasgow Coma Scale (GCS) score of 8 or less. The APACHE II tool is a widely used illness severity scoring system based on three variables; the worst physiological derangement recorded within the first 24 h of ICU admission, age and chronic health status (Knaus *et al*, 1985; Afessa *et al*, 2003).

### End points

ICU and long-term survival were analysed ‘by patient’. For those patients who were admitted to ICU more than once, outcome and survival were analysed from their last ICU admission. Long-term outcome was assessed by mortality rates of ICU survivors at latest follow-up. Cause of death was identified from the medical notes and/or post mortem examination results where available. Other standard endpoints analysed were TRM (non-relapse mortality related directly to the Allo-HSCT) and relapse of the underlying haematological malignancy (confirmed by clinical findings and histopathological examination).

### Statistical analysis

Effects on survival during an ICU admission were compared by logistic regression. Overall survival was calculated by Kaplan–Meier analysis and comparisons of survival were made by the log-rank test (for binary variables) and Cox regression (for categorical variables with more than two categories). *P* values < 0·05 were considered statistically significant. Ninety-five per cent confidence intervals (95% CI) are shown for survival analyses. Multivariate analysis of overall survival was by Cox regression. The multivariate model used included all terms significant or trend (*P* < 0·1) to univariate analysis and then eliminated non-significant terms.

## Results

### Patient demographics and transplantation type

Patient demographics, disease characteristics and transplant details are summarized in Table I. A total of 164 Allo-HSCT patients were included in the study. Median follow-up of all surviving patients was 43 months (range 4–101), with a median follow-up of 30 months (12–101) for MA patients and 45 months (9–69) for RI patients (*P* = 0·7111).

**Table I:** Characteristics of patients having one or more ICU admission after allogeneic HSCT (*n* = 164)

Characteristic	MA*N* = 127	RI*N* = 37	*P* value
Sex			
Male	48 (38%)	17 (46%)	0·446
Female	79 (62%)	20 (54%)	
Age at (last) admission			
Median (range), years	39 (11–60)	50 (23–66)	**0·0001**
Time since transplant			
Median (range), days	32 (1–476)	69 (6–1989)	**0·0007**
Diagnosis			
ALL	34 (27%)	0	**0·0001**
AML	36 (28%)	0	
NHL	20 (16%)	20 (54%)	
HL	5 (4%)	6 (16%)	
CLL	0	4 (11%)	
MF	1 (1%)	3 (8%)	
CML	14 (11%)	3 (8%)	
MDS	6 (5%)	0	
MM	11 (9%)	1 (3%)	
Donor stem cell source			
Sib	66 (40%)	16 (10%)	
UD	61 (37%)*	21 (13%)†	
Number of ICU admissions			
1	99 (78%)	26 (70%)	0·592
2	17 (13%)	8 (22%)	
3	8 (6%)	2 (5%)	
4	3 (2%)	1 (3%)	
Duration of ICU stay			
Median (range) days	4 (0–52)	6 (0–21)	0·4451

ICU, intensive care unit; HSCT, haematopoietic stem cell transplantation; ALL, acute lymphoblastic leukaemia; AML, acute myeloid leukaemia; NHL, non-Hodgkin lymphoma; HL, Hodgkin lymphoma; CLL, chronic lymphocytic leukaemia; MF, myelofibrosis; CML, chronic myeloid leukaemia; MDS, myelodysplasia; MM, multiple myeloma; Sib, matched sibling donor; UD, Unrelated Donor; RI, Reduced Intensity.

* Includes 3 Haploidentical donors.

† Includes 1 umbilical cord blood donation.

The median age of patients at last admission to ICU was 41 years (range 11–66) and the recipients of RI Allo-HSCT were significantly older at 50 years (range 23–66) compared to MA Allo-HSCT patients at 39 years (range 11–60), *P* = 0·0001. The cohort included 12 adolescent patients aged 11–17 years. Sixty percent of patients were male, with no significant difference between the MA and RI groups. Transplants were performed for a range of haematological malignancies. One hundred and twenty-seven patients (77%) were admitted to ICU following MA Allo-HSCT and 37 (23%) following RI Allo-HSCT.

As per our standard clinical practice, the majority of patients underwent T cell depleted (TCD) Allo-HSCT. RI Allo-HSCT patients were conditioned with fludarabine, melphalan and *in vivo* alemtuzumab (FMC), whilst MA Allo-HSCT patients were conditioned either with total body irradiation (TBI), fludarabine, cyclophosphamide and *ex vivo* alemtuzumab. A small number of MA Allo-HSCT patients were conditioned with TBI and cyclophosphamide or etoposide (T-replete). GvHD prophylaxis was ciclosporin alone, at 3 mg/kg per day starting on day −1 with a target level of 150–200 ng/ml (additional short-course methotrexate was given to patients receiving T-replete MA transplants). In the absence of GvHD, ciclosporin was tapered from 3 months after transplantation. Acute and chronic GvHD were graded according to standard consensus criteria. Supportive care was given according to local policy and escalated as clinically appropriate. Patients at risk of cytomegalovirus re-activation were monitored by weekly quantitative polymerase chain reaction and pre-emptively treated with intravenous ganciclovir or foscarnet.

The median time from transplant to ICU admission for all admissions (*n* = 213) was 42 d (range −5 d to 5·5 years), with recipients of MA Allo-HSCT being admitted to ICU earlier than RI-conditioned patients (median 32 d compared to 50 d, respectively, *P* = 0·0007).

During the study period, 552 patients underwent an Allo-HSCT procedure; of these, 164 (30%) required 1 or more ICU admission. Of the 164 patients, 125 (76%) had one single ICU admission, and 39 (24%) required 2–4 admissions.

Despite an older median age of patients undergoing RI Allo-HSCT compared to MA conditioning (age at time of transplant: 47 vs. 36 years, respectively, *P* < 0·0001) only 37/218 (17%) RI Allo-HSCT recipients were admitted to ICU at least once compared to 127/334 (38%) following MA Allo-HSCT (*P* < 0·001). The ICU admission rate for the 1119 autologous stem cell transplants carried out at our centre during the same period was 5%.

### Indications for and characteristics of ICU admissions

Individual ICU admission characteristics including the reason for admission, duration of ICU stay, number of organ systems supported during admission, APACHE II score and other laboratory data are detailed in Table II.

**Table II:** Characteristics of all Allo-HSCT related ICU admissions (*n* = 213)

		*N*	%
Characteristic		Median	Range
Duration of ICU admission	Days	4	1–52
		*N*	%
Reasons for admission (not mutually exclusive)	Sepsis	142	67
	Respiratory	117	55
	Renal	27	13
	Haemodynamic	26	12
	Neurological	6	3
	Observation	4	2
	Post-operative	3	1
	Liver failure	3	1
	Other*	6	3
	Unknown	3	1
Organ support (not mutually exclusive)	Non-Invasive ventilation	74	35
	Mechanical ventilation	107	50
	Inotropes	96	45
	Renal	56	26
	No support	44	21
Indices on admission		Median	Range
	Neutrophils (× 10^9^/l)	<0·5	0–18
	Platelets (× 10^9^/l)	30	0–649
	Urea (mmol/l)	9·9	1·8–45
	Bilirubin (mmol/l)	31·5	3–1081

APACHE II score		Median	Range
		23	4–51
		*N*	%
	4–10	5	2
	11–20	64	31
	21–30	102	49
	31–40	34	16
	>40	2	1
	Not available	6	3

Allo-HSCT, allogeneic haematopoietic stem cell transplantation; ICU, Intensive Care Unit; APACHEII, Acute Physiology and Chronic Health Evaluation Score.

* Other reasons for admission (*n* = 6): post-cardiac arrest (*n* = 2), poisoning, for open lung biopsy, post-liver biopsy, for endoscopy.

The total number of ICU admissions described in Table II was 213. The most frequent indication for admission were sepsis (67%) and/or respiratory failure (55%). Indications for admission were not mutually exclusive and patients were often admitted with multiple factors. Ventilatory support was required in 132 (62%) admissions. Mechanical ventilation (MV) was required in a total of 107 admissions (50%), including 49 (23%) where MV followed non-invasive ventilation (NIV). During a further 25 admissions, NIV alone was required (19%). In 44 admissions (21%) no mechanical or vasoactive drug organ support was instituted.

Multilevel model stratification by patient was used to assess the impact of conditioning intensity on median admission duration and APACHE score on admission. The median duration of an ICU admission was 4 d (range 1–52), being 4 d for the MA Allo-HSCT group (range 0–52) and 6 d for the RI All-HSCT group (range 0–21), *P* = 0·345. The median APACHE II score was 23 (range 4–51). There was no significant difference in APACHE II score between the RI conditioning and MA conditioning recipients (median 22 vs. 23, respectively, *P* = 0·646).

### Short-term outcome: ICU survival by patient

When short-term outcome by patient (*n* = 164) was considered, 53 patients (32%) survived ICU (Table III). For patients admitted to ICU more than once, median follow-up and survival endpoints were measured at discharge from the final admission.

**Table III:** ICU survival and causes of death for all 164 Allo-HSCT patients

	%	*N*
ICU survival		
Total patients		164
Yes	32	53
No	68	111
ICU survival		
Conditioning		
MA	27	34
RI	51	19
Cause of death in ICU (Not mutually exclusive)		
Total deaths		111
Sepsis	35	39
MOF	27	30
Respiratory	39	44
Invasive fungal infection	5	6
GvHD	5	5
Other*	14	15

ICU, Intensive Care Unit; Allo-HSCT, allogeneic haematopoietic stem cell transplantation; MOF, Multi Organ Failure; GvHD, Graft-versus-host disease; RI, Reduced Intensity; MA, Myeloablative.

* Other causes of death on ICU = relapse (*n* = 2), cardiac arrest (*n* = 2), gastrointestinal bleed (*n* = 2), pulmonary haemorrhage (*n* = 1), intracerebral haemorrhage (*n* = 1), hepatic failure (*n* = 1), graft failure (*n* = 1), Post-transplant lymphoproliferative disorder (*n* = 1), left ventricular perforation during pericardial drain insertion (*n* = 1), ciclosporin-related thrombotic thrombocytopenic purpura (*n* = 1).

The causes of death on ICU were recorded in Table III. Pneumonia and other respiratory disorders combined (pneumonitis, lung injury and acute respiratory distress syndrome – ARDS) were the cause of death in 44 patients (39%). Overwhelming sepsis was the cause of death in 39 patients (35%). The identifiable cause of death on ICU is often multi-factorial and as such, the causes of death were not mutually exclusive.

A number of variables affecting ICU survival were considered in more detail and are shown in Table IV. Despite MA Allo-HSCT patients being admitted to ICU significantly earlier in their transplant course, compared to recipients of RI Allo-HSCT, no significant effect on ICU survival of proximity of ICU admission to stem cell return (Day 0) was observed (*P* = 0·161).

**Table IV:** Variables influencing ICU survival (*n* = 164 patients)

Effects on ICU survival (univariate analysis)	OR (95% CI)	*P* value	*N*
Reason for admission			
Sepsis	0·708 (0·367–1·367)	0·304	27/93 (29%)	
Respiratory	0·543 (0·281–1·053)	0·071	24/91 (26%)	
Renal	0·422 (0·135–1·315)	0·137	4/22 (18%)	
Haemodynamic	0·451 (0·144–1·415)	0·172	4/21 (19%)	
Neurological	0·692 (0·070–6·818)	0·753	1/4 (25%)	
Donor			
UD *versus* Sib	0·946 (0·491–1·820)	0·867	26/79 (33%) vs. 27/58 (32%)	
Conditioning			
RI *versus* MA	2·887 (1·357–6·142)	0·006	19/37 (51%) vs. 34/127 (27%)	
Organ support			
NIV	0·390 (0·178–0·856)	0·019	13/50 (21%)	
MV	0·093 (0·042–0·208)	<0·001	11/83 (12%)	
Inotropes	0·176 (0·083–0·373)	<0·001	13/87 (15%)	
Renal	0·243 (0·100–0·590)	0·002	7/52 (13%)	
No ventilatory support *versus* NIV only *versus* MV ± NIV	0·240 (0·147–0·389)	<0·001	23/33 (70%) vs. 7/37 (37%) vs. 11/94 (12%)	
Number of organs supported			
0 vs. 1 vs. 2 vs. 3	0·385 (0·273–0·541)	<0·001	33/33/45/45
Neutrophil count	1·013 (0·931–1·102)	0·768	
Platelet count	1·006 (1·000–1·011)	0·062	
Urea	0·930 (0·885–0·977)	0·004	
Bilirubin	1·000 (0·996–1·003)	0·978	
APACHE II	0·930 (0·883–0·980)	0·006	
Duration of ICU stay	0·952 (0·907–0·998)	0·042	
Duration of MV	0·982	0·694	
Combined duration of NIV ± MV	0·945	0·170	
Year of ICU admission	1·102	0·072	
Number of ICU admissions	0·976 (0·611–1·559)	0·920	

Effects on ICU survival (multivariate analysis)	OR (95% CI)	*P* value
MV	0·071	<0·001
Urea	0·931	0·007
Conditioning intensity	3·27	0·023

ICU, intensive care unit; OR, odds ratio; 95% CI, 95% confidence interval; UD, unrelated donor; Sib, matched sibling donor; RI, reduced intensity; MA, myeloablative; NIV, non-invasive ventilation; MV, mechanical ventilation; APACHEII, Acute Physiology and Chronic Health Evaluation Score.

The number of ICU admissions did not affect probability of surviving to discharge (*P* = 0·920), nor was it significantly different between RI and MA patients. There was, however, a trend to poorer prognosis for those survivors having had >1 admission to ICU (*P* = 0·069).

ICU survival was significantly better in patients admitted after RI conditioning compared to MA conditioning (51% vs. 27%, OR 2·878; *P* < 0·01).

Further variables significantly affecting ICU survival by patient on univariate analysis are detailed in Table IV. Intensity of pre-transplant conditioning regimen, requirement for organ support – including NIV alone, APACHE II score, serum urea, and duration of ICU admission were all significantly associated with adverse outcome. Interestingly, in our study, duration of MV (and MV/NIV) was not associated with a poorer outcome.

On multivariate analysis, MV and raised serum urea at the time of admission remained associated with a poor short-term outcome (*P* < 0·001 and *P* = 0·007, respectively). Importantly, conditioning type also remained significant; patients who underwent RI Allo-HSCT had a significantly better ICU survival rate compared to those who received MA conditioning (*P* = 0·023) (Table IV).

It is likely that analysis by last admission inflates the mortality incidence as earlier ICU admissions, which were survived, were not included and the fatal admission is always the last. However, no differences were observed on univariate analysis when outcome was analysed by first admission to ICU (data not shown). In addition, as indicated in Table I, the number of admissions per patient were statistically similar between the MA and RI groups (*P* = 0·592).

### Long-term outcome

**Fig. 1:**
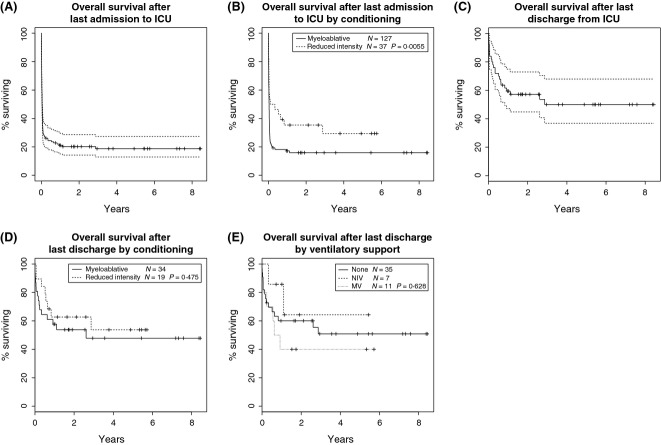
(A) Kaplan–Meier survival curve for all patients admitted to the intensive care unit (ICU) (including deaths on ICU), *n* = 164. (B) Kaplan–Meier survival curve for all patients: Overall Survival after last admission by transplant conditioning intensity (Survival curves compared using log-rank test). (C) Kaplan–Meier survival curve for ICU survivors: Overall Survival after last discharge from ICU, *n* = 53. (D) Kaplan–Meier survival curve for ICU survivors: Overall Survival by conditioning (Survival curves compared using log-rank test). (E) Kaplan–Meier survival curve for ICU survivors: Overall Survival by ventilatory support on ICU (Survival curves compared using log-rank test).

The overall survival rate at 1 and 5 years from time of ICU admission for the whole cohort (i.e. including deaths in ICU), was 19% and 17%, respectively (Fig. 1A).

As described above, 53/164 (32%) patients survived ICU admission. For these patients, subsequent long-term survival was excellent with 1- and 5-year survival rates of 61% and 51%, respectively, at a median follow-up for all patients of 43 months (range 8–101 months) (Fig. 1C). Median follow-up for MA Allo-HSCT patients was 30 months (range 1–101) and 45 months (9–69) for RI Allo-HSCT patients. No significant difference in causes of death was observed (TRM or relapse) between the MA and RI groups. TRM at 5 years was 40% (95% CI: 21–58) in the MA Allo-HSCT group and 35% (95% CI: 14–58) in the RI Allo-HSCT group, *P* = 0·566. Similarly, relapse mortality at 5 years was 11% for both groups (95% CI: 3–25 and 2–29, respectively), *P* = 0·975.

The improved survival for RI recipients admitted to ICU remained significant with subsequent long-term follow-up (*P* = 0·0055) (Fig. 1B) but this was due solely to differences in immediate ICU survival (Fig. 1D). Similarly, requirement for ventilatory support did not independently impact on subsequent long-term survival in those who survived ICU (Fig. 1E).

The long-term survival of ICU survivors was equivalent to that observed for Allo-HSCT patients who did not require admission to ICU peri- or post-transplant (data not shown).

## Discussion

The admission of Allo-HSCT patients to intensive care remains contentious based on published outcomes in this patient group which are generally poor, even with more recent studies demonstrating improvements (Naeem *et al*, 2006; Pène *et al*, 2006; Depuydt *et al*, 2011). There is a perception that transfer to ICU may be futile and long-term outcome poor, even if patients were to survive a critical illness episode. In our study, the ICU survival rate following Allo-HSCT, measured at the time of final ICU admission for those admitted more than once, was 32%; this compares favourably with other published data in which ICU survival was 30–51% (Pène *et al*, 2006; Lim *et al*, 2007; Bokhari *et al*, 2010; Depuydt *et al*, 2011).

During the study period, 30% of patients undergoing Allo-HSCT required ICU admission with a significantly lower admission rate following RI than MA conditioning, despite the significantly older median age of RI recipients. Published admission rates (11–40%) and reasons for admission (predominantly respiratory failure and/or sepsis) are comparable, as is the severity of illness (Afessa *et al*, 1992; Faber-Langendoen *et al*, 1993; Rubenfeld & Crawford, 1996; Jackson *et al*, 1998; Paz *et al*, 1998; Price *et al*, 1998; Soubani *et al*, 2004; Naeem *et al*, 2006). Invasive ventilation was required in 50% of admissions and the APACHE II score was >20 in 65% of admissions. For comparison, the median APACHE II score for all UK ICU admissions is 16·5 (Harrison *et al*, 2004), and was 20–25 in international trials of sepsis in ICU (Bernard *et al*, 2001; Brunkhorst *et al*, 2008).

The factors most consistently associated with poor ICU outcome in HSCT patients are multi-organ failure and mechanical ventilation (Afessa *et al*, 1992; Rubenfeld & Crawford, 1996; Hinds *et al*, 1998; Soubani *et al*, 2004), raised bilirubin (Jackson *et al*, 1998; Price *et al*, 1998; Afessa *et al*, 2003), increasing age (Faber-Langendoen *et al*, 1993; Epner *et al*, 1996; Hinds *et al*, 1998; Darmon *et al*, 2002; Rabe *et al*, 2004), and increased APACHE or Sequential Organ Failure Assessment (SOFA) scores (Knaus *et al*, 1985; Afessa *et al*, 2003; Depuydt *et al*, 2004; Cornet *et al*, 2005; Cherif *et al*, 2007). Our results corroborate these findings with the notable exception of elevated bilirubin, which was not significantly associated with a negative ICU outcome. This may reflect the fact that the majority of patients underwent T-cell depleted Allo-HSCT, with a consequently low incidence of GvHD. As with other studies (Paz *et al*, 1993, 1998; Epner *et al*, 1996; Rubenfeld & Crawford, 1996; Lim *et al*, 2007), we found that ICU survival in our cohort of patients was affected adversely by the number of organs supported during ICU admission and the need for ventilatory support (21% compared to 87% when no ventilatory support was required). Unlike previous studies, we identified NIV as a statistically significant independent predictor of ICU survival, again reflecting the prognostic importance of respiratory failure.

RI conditioning regimens have reduced TRM and permitted the use of potentially curative transplants in older patients (Chakraverty *et al*, 2002; Bacigalupo *et al*, 2004; Morris & Mackinnon, 2005; Craddock, 2008; Thomson *et al*, 2010; Peggs *et al*, 2011). In the UK the majority of allogeneic transplants performed in adults now utilize RI conditioning (K. Kirkland, BSBMT Data Registry, personal communication). The effect of this on ICU survival has not previously been clearly reported because RI Allo-HSCT recipients accounted for too few patients in previous studies to draw meaningful conclusions about the impact of conditioning intensity on ICU survival (Pène *et al*, 2006; Lim *et al*, 2007; Depuydt *et al*, 2011). Our study included 37 (23%) patients who had undergone RI conditioning and we found that conditioning intensity significantly influenced ICU survival despite there being no significant difference in median APACHEII score or requirement for MV.

There few published large studies concerning long-term survival of Allo-HSCT patients after ICU discharge. We observed a 5-year overall survival of 51% for patients surviving ICU admission. This important finding from a large study supports the position that critically ill Allo-HSCT patients should be considered for ICU support, as excellent long-term survival is potentially achievable.

Critical care support and management have improved over recent years as a result of early recognition and intervention in sepsis (Rivers *et al*, 2001), improved understanding and management of ARDS, increased use of NIV (Azoulay *et al*, 2001), and a move to admit sick patients to the ICU before organ failure becomes firmly established (Bokhari *et al*, 2010). The improved survival rates in more recent studies may be in part due to these changes, and in part to better patient selection and management of the terminal phase of illness on the ward rather than in ICU (Naeem *et al*, 2006). Year of admission to ICU had a trend to significance only in the univariate analysis performed on our study cohort (*P* = 0·07), but this was non-significant on subsequent multivariate analysis. It is likely that much larger studies would be required to demonstrate such an effect ‘of era’ on improving outcomes, as changes in supportive care are confounded by changes in transplant protocols. Clearly, the decision to admit to ICU requires an accurate assessment of the underlying condition, prognosis and reversibility of illness (Azoulay *et al*, 2001).

Application of the findings from our retrospective single centre study to different patient cohorts and different centres should be performed with caution at present. However, ours is one of the largest studies examining the survival of patients admitted to ICU following Allo-HSCT with 213 consecutive admissions over an 11·5-year period. It includes long-term follow-up data beyond 1 year post-ICU discharge, and this demonstrates excellent subsequent long-term survival. It is also one of the first studies looking at ICU survival after RI allogeneic transplants.
